# Developmental expression of three small GTPases in the mouse eye

**Published:** 2007-07-13

**Authors:** Dianne C. Mitchell, Brad A. Bryan, Jin-Ping Liu, Wen-Bin Liu, Lan Zhang, Jia Qu, Xiangtian Zhou, Mingyao Liu, David W. Li

**Affiliations:** 1 Institute of Biosciences and Technology, Texas A&M University System Health Science Center, Houston, TX; 2 Department of Biochemistry & Molecular Biology, College of Medicine, University of Nebraska Medical Center, Omaha, NE; 3 Key Laboratory of Protein Chemistry & Developmental Biology of Education Ministry of China, College of Life Sciences, Hunan Normal University, Changsha, Hunan, China; 4 School of Ophthalmology and Optometry, Wenzhou Medical College, Wenzhou, Zhejiang, China; 5 Department of Ophthalmology and Visual Sciences, College of Medicine, University of Nebraska Medical Center, Omaha, NE

## Abstract

**Purpose:**

The small GTPases function as "molecular switches" by binding and releasing GTP to mediate downstream signaling effects. The Rho-family of GTPases is central in modulating cell differentiation and cytoskeletal changes. Since eye development requires comprehensive morphogenetic movements and extensive cellular differentiation, we hypothesize that different small GTPases may play important roles during morphogenesis of eye development. To explore this possibility, we examined the expression patterns of three major Rho-GTPases: RhoA, Rac1, and Cdc42 in embryonic, postnatal (one day after birth), and adult (two-month old) mouse eye.

**Methods:**

Various ocular tissues were collected from embryonic, postnatal, and adult C57BL/6 mice. Western blots were conducted using total proteins extracted from cornea, retina, lens epithelial cells, and lens fiber cells of the adult mice or different fractions of rat lenses. Immunohistochemistry (IHC) was performed with 6 μm thick sections cut through the eye ball region of 11.5 pc, 14.5 pc, 17.5 pc, postnatal, and adult mice. Parallel controls were run using the rabbit preimmune and GTPase-specific antibodies blocked with saturating levels of corresponding peptide antigen.

**Results:**

In the embryonic mouse eye, RhoA and Cdc42 expressions were initially detectable in all three compartments at 11.5 pc. However, Rac1 became easily detectable in these compartments at 14.5 pc. Increased levels of RhoA, Rac1, and Cdc42 were detected in the three compartments at 17.5 pc and the strongest signals for RhoA, Rac1, and Cdc42 were observed in the primary lens fiber cells at 17.5 pc. In the postnatal mouse eye, the three small GTPases were significantly expressed in both endothelial and epithelial cells of mouse cornea, epithelial cells of the ocular lens, photoreceptors, horizontal/amacrine/Muller's cells, and some ganglian cells of the retina. Much lower level of expression was observed in the corneal stroma fibroblasts, lens fiber cells, and the inner and outer plexiform layers of the mouse retina. In the adult mouse eye, all three Rho-GTPases were expressed in corneal epithelial cells and retina. However, only RhoA protein was detected in corneal endothelial cells and Rac1 protein detected in the ocular lens.

**Conclusions:**

The strong expression of the three small GTPases in the cornea, lens, and retina of mouse eye at embryonic 17.5 pc and postnatal stage suggests their important functions for the morphogenesis of the different compartments of the mouse eye. Particularly, high levels of expression of RhoA, Rac1, and Cdc42 in embryonic lens fiber cells suggest their involvement in differentiation of primary lens fiber cells. In the adult mouse eye, all three Rho-GTPases seem to be involved in differentiation of corneal epithelial cells and retina, however, RhoA alone may be required for endothelial cell differentiation and Rac1 likely plays an important role in supporting continuous lens growth and maintenance of lens transparency.

## Introduction

The small molecule GTPases function as "molecular switches", mediating extracellular signaling events to downstream intracellular effectors by cycling between GDP-bound inactive and GTP-bound active forms upon stimulation. The Rho family of small molecule GTPases, which among them RhoA, Rac1, and Cdc42 are most highly characterized, are implicated in numerous cellular functions including cytoskeletal actin organization [1], cell growth, cell migration [2], cell adhesion [1], vesicular trafficking, and differentiation [3,4]. These different Rho family GTPases regulate the formation of distinct structural elements; Rho induces formation of actin stress fibers and cell contacts while Cdc42 regulates filopedia formation and Rac1 regulates membrane ruffling [4]. Although, once thought to be ubiquitously and uniformly expressed in mammalian tissues, differential expression patterns of these proteins have been shown in the brain [5] as well as throughout retinal development in the chicken [6]. Differences in expression of these Rho family proteins in neuronal cells have proven to be essential for proper neurite outgrowth, as each member prompts distinct effects on the growth of neuronal processes and axons. It is possible that differential expression patterns of these proteins in other tissues are equally important for the growth and perpetuation of other cells and systems [7,8].

The vertebrate eye develops through a complex process of morphogenesis which is modulated by various signaling transduction pathways and governed by different families of regulators. Among these regulators are the small molecule GTPases. Various members of the Rho family of small molecule GTPases were previously found to be present in both the ocular lens and the retina [6,9]. To further explore the potential functions of the small molecule GTPases in regulating the development of different compartments of the eye, we have examined the expression patterns of RhoA, Rac1, and Cdc42 throughout mouse eye development. Our results are the first to reveal the expression patterns of these small molecule GTPases in cornea and of the temporal differences in expression patterns of RhoA, Rac1, and Cdc42 in the ocular lens from embryonic stages to the postnatal stage (one day after birth) to adult (two months old) stage of the mouse eye. In addition, our studies provide comparative information on the expression patterns of RhoA, Rac1, and Cdc42 in the developing retina and in the adult retina. Together, our studies provide important information regarding the functions of RhoA, Rac1, and Cdc42 in regulating morphogenesis of the three compartments of the eye: retina, lens, and cornea.

## Methods

### Animals

The C57BL/6 mice used in the experiments were obtained from Jackson Laboratory. The four week old Sprague-Dawley rats were purchased from Harlan. Animals were maintained in 12 h of light/dark cycles and fed normal diet and water. Mice and rats used in this study were handled in compliance with the "Guide for the Care and Use of Laboratory Animals".

### Preparation of embryo sections

Ocular tissues from an embryonic mouse at 11.5 pc, 14.5 pc, and 17.5 pc stages, from postnatal (one day after birth) mice, and from adult (two month old) mice were fixed in zinc-formalin fixative and processed by Excalibar Pathology (Moore, OK). The slides were deparaffinized with xylene, rehydrated, and bathed in 3% H_2_O_2_ at 37 °C to quench the endogenous peroxidase as well as unmask protein antigens by steaming in citrate solution for 45 min.

### Immunohistochemistry

Immunohistochemistry (IHC) of paraffin embedded tissues from mice was performed using the ABC-Staining system according to manufacturer's instructions (Santa Cruz Biotechnology, Santa Cruz, CA). All sections were counterstained with Harris Modified Hematoxylin with acetic acid (Fisher Scientific, Pittsburgh, PA). Both monoclonal (anti-RhoA and -Cdc42) and polyclonal (anti-Rac1) antibodies were obtained from Santa Cruz Biotechnology. Primary antibodies were applied at a 1:100 dilution for IHC. Control IHC experiments were performed using the rabbit pre-immune serum and GTPase-specific antibodies blocked with saturating levels of corresponding RhoA, Rac1, or Cdc42 peptide antigen.

### Extraction of total proteins from the cornea, lens, and retina from adult mouse

To extract total proteins from the cornea, retina, or the whole lens, different parts of the mouse eye tissues were dissected out, grinded under liquid nitrogen to make a fine powder, and suspended in protein extraction buffer (50 mM Tris, 150 mM NaCl, 1 mM EDTA, 1% NP-40, 1 mM PMSF, 10 μg/ml leupeptin, 10 μg/ml aprotinin, and 1 mM sodium orthovanadate) [10]. The resuspended samples were passed through an 18.5 G and 23.5 G needle for ten times each and then centrifuged at 4 °C with 14,000 rpm for ten min. Both supernatant and pellet were saved. The pellet was resuspended in additional cell lysis buffer. An equal amount of SDS sample buffer was added into both the supernatant and the resuspended pellet fraction. The resuspended pellet was initially examined to ensure that no small GTPases were present. The supernatant was subjected to western blot analysis as described below. To further confirm that the expression patterns of RhoA, Rac1, and Cdc42 in the ocular lens, both epithelial and fiber cells from mouse lenses were dissected into Eppendorf tubes and were homogenized ten times in the above extraction buffer containing pepstatin (5 μg/ml), benzamidine (50 μg/ml), glycerophosphate (10 mM), and pyrophosphatase (10 mM) with a dounce homogenizer carrying a loose fitting pestle. The homogenized samples were centrifuged 1000x g in JA-14 rotor to collect the plasma membrane from the pellet [11]. Then additional extraction buffer was added into the pellet and the mixture was homogenized 15 times in a Teflon homogenizer. The mixture was then passed through an 18.5 G needle ten times and a 23.5 G needle ten times before adding an equal amount of 2X SDS sample buffer into the sample. These protein samples were then used for western blot analysis and the results obtained were consistent with those revealed by the procedures described above.

### Isolation of total proteins from lens epithelia and different fractions of fiber cells in rat lenses

After dissection of rat lenses from the intact eyeballs, the capsular epithelium from each lens was removed and transferred into a prechilled Eppendorf tube containing protein extraction buffer. The deepithelium lens was immediately transferred into a 10 ml beaker for extraction of different layers of fiber proteins as previous described [12,13]. For extraction of total proteins, the epithelia were homogenized on ice with an Eppendorf tube micropestle (Brinkmann Instruments, Inc., Westbury, NY). Various fractions of fiber cells were homogenized for 20 strokes with a Kimax glass homogenizer. The homogenates of both epithelial and fiber cells were then centrifuged at 10,000x g for 20 min at 4 °C. The supernatant of each sample was collected in aliquots, frozen with liquid nitrogen, and then stored at -80 °C for further analysis.

### Western blotting analysis

For each sample, the protein concentration was determined as previously described [14]. Western blot analysis of total proteins was conducted as described before [12,13]. Briefly, 100 μg of total proteins in each sample were resolved by 15% SDS-polyacrylamide gel. The protein blots were blocked with 5% milk in TBS (10 mM Tris-HCl, pH 8.0/150 mM NaCl) overnight at 4 °C, and were incubated with primary antibodies as described above. Following incubation with the primary antibody, the membrane was exposed to a horseradish peroxidase-conjugated secondary antibody, subjected to SuperSignal West Pico Chemiluminescent reagent (Pierce Biotechnology, Inc., Rockford, IL), and exposed to film. A single protected band at approximately 21 kDa was observed for each GTPase-specific antibody used.

### Reverse transcription-linked polymerase chain reaction

Reverse transcription was conducted using a kit from Gibco BRL (number 18085-019; Gaithersburg, MD) as previously described [15,16]. Briefly, 3 μg of total RNA were used in a total reaction volume of 25 μl. For PCR amplification, the following primers were used: β-actin, 5'-GTG GGG CGC CCC AGG CAC CA-3' (forward) and 5'-CTC CTT AAT GTC ACG CAC GAT TTC-3' (reverse); mouse RhoA (316 bp), 5'-TGA AAA CTA TGT GGC GGA TAT CG-3'(forward) and 5'-TCT GCT TCT TCA GGT TTA ACC GG-3' (reverse); mouse RhoB (375 bp), 5'-AGG ACT ACG ATC GTT TAC GGC C-3' (forward) and 5'-CAG CCA TTC TGG GAT CCG TAG-3'(reverse): mouse Rac1 (485 bp), 5'-CTG CCT GCT CAT CAG TTA CAC G-3'(forward) and 5'-GGA CAG AGA ACC GCT CGG ATA-3' (reverse); and mouse Cdc42 (315 bp), 5'-GTT CGT TAC CGA GAG CGT ATC A-3'(forward) and 5'-GCT TGG TTA CCA CAA CGA TAG C-3'(reverse). Two microliters of the reverse transcription reaction mixture were used for PCR reactions. For PCR, β-actin and RhoA, RhoB, Rac1, or Cdc42 primers were added in pair at the same time for a total of 30 cycle amplification. Each cycle was run with the following program: denaturing at 94 °C, 30 s; annealing at 52 °C, 30 s; and chain extension at 72 °C, one min. At the end of each reaction, the PCR products were separated by agarose gel (1.5%) electrophoresis and photographed under UV illumination.

## Results

### Expression of RhoA, Rac1, and Cdc42 in the cornea of the embryonic mouse eye

**Figure 1:**
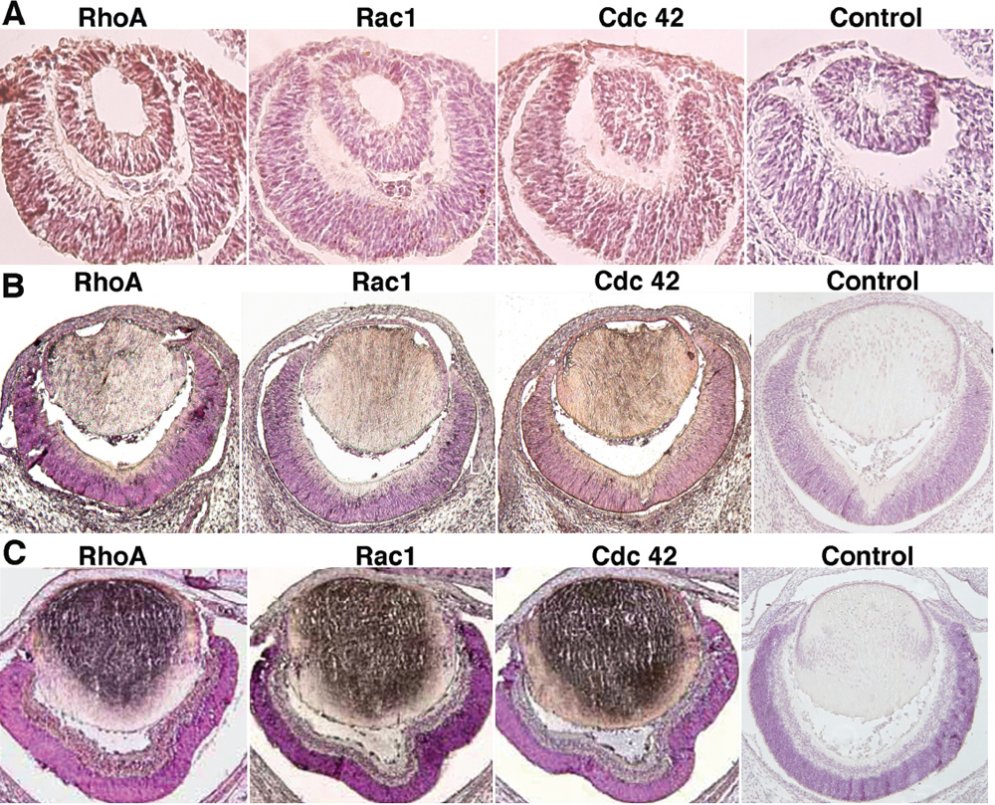
Immunocytochemistry analysis of the expression patterns of RhoA, Rac1, and Cdc42 during mouse eye development. **A**: Expression of RhoA, Rac1, and Cdc42 on day 11.5 pc mouse embryonic eye. RhoA was highly expressed in the peridermal cornea, lens vesicle and differentiating retina. Compared with RhoA, Cdc42 was expressed in a similar pattern in these compartments but the level was lower. Expression of Rac1 in the three compartments was detectable but in a much lower level in comparison to both RhoA and Cdc42. **B**: Expression of RhoA, Rac1, and Cdc42 on day 14.5 pc mouse embryonic eye. Expression of the three GTPases is elevated in the differentiating cornea, lens fibers, and the inner region of the neuroblastic layer. **C**: Expression of RhoA, Rac1, and Cdc42 on day 17.5 pc mouse embryonic eye. Note that strong expressions of RhoA, Rac1, and Cdc42 were observed in the epithelial and primary fiber cells of the embryonic lens. To a less degree, these small GTPases are expressed in the cortical fiber cells, corneal epithelial and endothelial cells, and differentiating retina.

**Figure 2:**
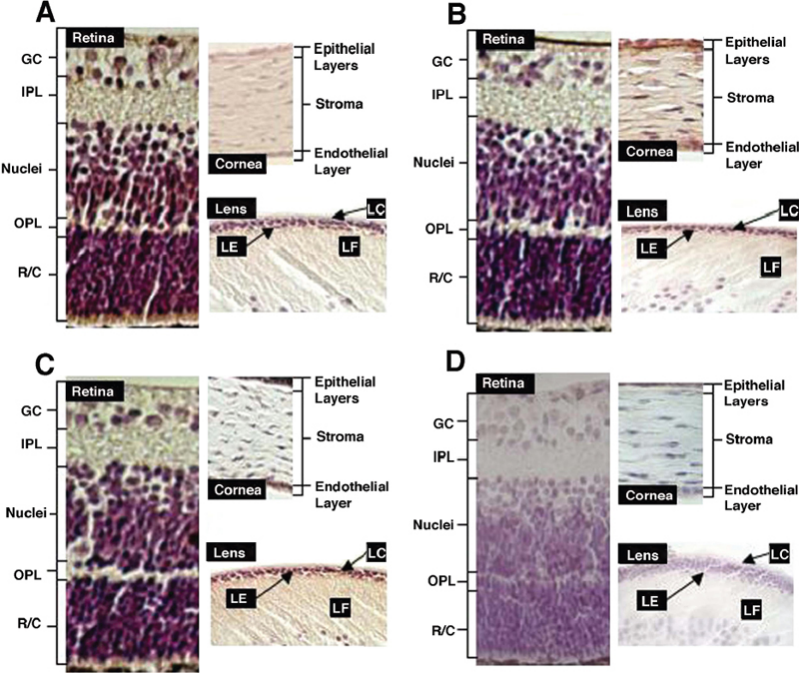
Immunocytochemistry analysis of the expression patterns of RhoA, Rac1, and Cdc42 in postnatal mouse eye. **A**: Expression of RhoA in different compartments. RhoA was highly expressed in the photoreceptors, the horizontal/amacrine/Muller cell layers, and in some ganglion cells. RhoA expression is relatively lower in other ganglion cells and plexiform layers. Corneal expression of RhoA was detectable in the endothelium and to a lesser degree in the epithelial cell layers. High level of RhoA expression was detected in lens epithelium and a much lower level of expression was seen in the fiber cells. **B**: Expression of Rac1 in different compartments. Like RhoA, Rac1 was highly expressed in the photoreceptors and the horizontal/amacrine/Muller cell layers as well as in some ganglion cells, but lower in other ganglion cells and plexiform layers. Corneal expression of Rac1 was highly detectable in both endothelial and epithelial cells. High level of Rac1 expression was detected in lens epithelium and to a less degree in the fiber cells. **C**: Expression of Cdc 42 in different compartments. Similar patterns of Cdc42 expression as Rac1 were observed in the three different compartments of postnatal mouse eye except that a very high level of Cdc42 was observed in the corneal epithelial cells. **D**: Control sections. The control sections were processed using the same procedures as the immunocytochemistry results shown in Figure 2A-Figure 2C, except that the primary antibodies were replaced with normal rabbit serum. LC: lens capsule; LE: lens epithelium; LF: lens fiber cells; GC: ganglion cells; IPL: inner plexiform; Nuclei: horizontal/amacrine/Muller cell layers; OPL: outer plexiform; R/C: rods and cones (photoreceptors).

At 11.5 pc of mouse development, the lens vesicle itself has formed and the ectoderm above the lens vesicle begins to differentiate into cornea (Figure 1A). Compared with the control (the rightmost panel), expression of RhoA and Cdc42 was clearly detected in this tissue (the leftmost panel and the third panel from the left of Figure 1A). In contrast, expression of Rac1 was nearly undetectable (the second panel from the left of Figure 1A). RhoA and Cdc42 continue to be expressed at 14.5 pc and by this stage of development, expression of Rac1 also begins to appear within the cornea (Figure 2B). At 17.5 pc, when the cornea differentiates into three different layers: endothelial cells, stroma and epithelial cells, expression of RhoA, Rac1, and Cdc42 was found more focused in the epithelial and endothelial cells (Figure 1C).

### Expression of RhoA, Rac1, and Cdc42 in the lens of the embryonic mouse eye

At 11.5 pc, strong expression of the RhoA was detected throughout the lens vesicle (the most left panel of Figure 1A). Expression of Cdc42 at this same stage was lower than that of RhoA (the third panel from the left of Figure 1A), while Rac1 expression was barely detectable (the second panel from the left of Figure 1A). In contrast to Rac1 expression, which was predominantly found in the differentiating primary fiber cells (the second panel of Figure 1B), both RhoA and Cdc42 expression at 14.5 pc was found in differentiating primary fiber cells as well as in the lens epithelium (the leftmost panel of Figure 1B and the third lane from the left of Figure 1B, respectively). Overall, expression of Cdc42 at 14.5 pc is relatively higher than either RhoA or Rac1 (Figure 1B). All three small molecule GTPases are more strongly expressed by 17.5 pc displaying heavy expression in the differentiating primary fiber cells and fibers in the subcortical region (Figure 1C). Enhanced expression of all three Rho GTPases was found in the primary fiber cells and to a lesser extent in the lens epithelium. In contrast, the expression of these small GTPases was much attenuated in the cortical region (Figure 1C).

### Expression of RhoA, Rac1, and Cdc42 in the retina of embryonic mouse Eye

In mouse retina, RhoA was also found expressed at 11.5 pc in the developing neuroretinal layers and the neuroblastic region (the leftmost panel of Figure 1A). During this same stage of development, the expression level of Cdc42 in retina was lower than RhoA and that of Rac1 was even lower (the third and the second panels from the left of Figure 1A, respectively). At stage 14.5 p.c., expression of RhoA was detected in the predicted photoreceptor zone and to a lesser degree in the retinal pigment epithelial cell layer (the leftmost panel of Figure 1B). Both Rac1 and Cdc42 reveal similar patterns of expression with Cdc42 expression being relatively stronger (Figure 1B). As development proceeds to 17.5 p.c., expression of all three small GTPases was strongly detected in both photoreceptor and pigment layers of the retina. Among the three small GTPases, Rac1 displayed the highest level of expression in the photoreceptor region (Figure 1C).

### Expression of RhoA, Rac1, and Cdc42 in the cornea of the postnatal mouse eye

Compared to the negative control (Figure 2D), RhoA was expressed in the endothelium and to a lesser degree in the epithelial cells and some stromal fibroblasts of the postnatal mouse (Figure 2A). Similarly, expression of Cdc42 was clearly detected in both endothelial and epithelial cells but hardly detectable in the stroma (Figure 2C). In contrast to both RhoA and Cdc42, strong levels of Rac1 expression were noted in the fibroblasts within the stroma as well as in both endothelial and epithelial cell layers of the postnatal mouse cornea (Figure 2B).

### Expression of RhoA, Rac1, and Cdc42 in the lens of the postnatal mouse eye

In the ocular lens, strong expression levels of RhoA, Rac1, and Cdc42 were detected in the lens epithelial cells (Figure 2A-C) of the postnatal mouse in comparison to the negative control (Figure 2D). In contrast, their expression levels in the fiber cells were substantially decreased (Figure 2A-C).

### Expression of RhoA, Rac1, and Cdc42 in the retina of the postnatal mouse eye

Compared with the negative control (Figure 2D), a strong expression level of RhoA was detected in the photoreceptors, the horizontal/amacrine/Muller's cells, and some ganglion cells of the postnatal mouse retina (Figure 2A). In the cellular bodies of most of the ganglion cells, and the inner and outer plexiform layers, a reduced level of RhoA was detected (Figure 2A). Expression of Rac1 and Cdc42 followed a similar pattern of RhoA but the expression levels were decreased in the retina of postnatal mouse eye (Figure 2B,C).

### Expression of RhoA, Rac1, and Cdc42 in the lens of the adult mouse eye

**Figure 3:**
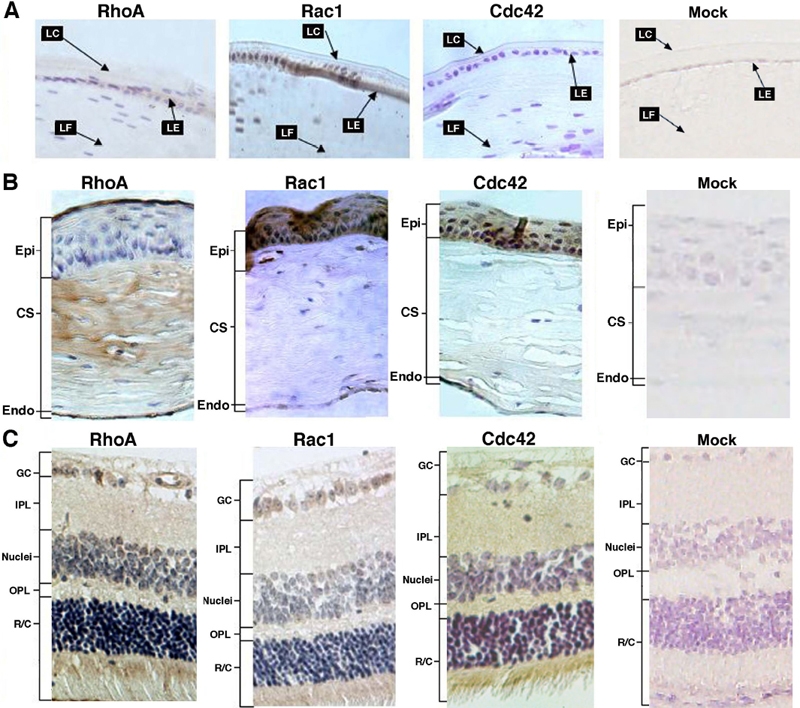
Immunocytochemistry analysis of the expression patterns of RhoA, Rac1 and Cdc42 in adult mouse eye. **A**: Expression of the three small GTPases in the adult mouse lens was investigated. While Rac1 was detected in both lens epithelium and fiber cells, expression of RhoA and Cdc42 were barely detectable in the adult mouse lens. Compared with the control, the nuclei of both epithelial and fiber cells of mouse lens displayed higher background. LC: lens capsule; LE: lens epithelium; LF: lens fiber cells. **B**: Expression of the three small GTPases in the adult mouse cornea was followed. Strong RhoA expression was detected in the top layer of corneal epithelial and endothelial cells. Some RhoA was detected in fiberoblast cells. Expressions of Rac1 and Cdc42 were strong in the epithelial layers but relatively lower in the endothelial cell. No obvious staining was seen in the corneal stroma. Epi: corneal epithelial cell layers; CS: corneal stroma; Endo: corneal endothelial cell layers. **C**: Expression of the three small GTPases in the adult mouse retina was investigated. Both RhoA and Cdc 42 were highly expressed in the photoreceptors, the horizontal/amacrine/Muller cell layers, and in some ganglion cells. In contrast, strong expression of Rac1 was only observed in the photoreceptors. GC: ganglion cells; IPL: inner plexiform; Nuclei: horizontal/amacrine/Muller cell layers; OPL: outer plexiform; R/C: rods and cones (photoreceptors). Mock sections in the rightmost panels of **A**, **B**, and **C** were processed in the same way as the immunocytochemistry results shown for RhoA, Rac1, and Cdc42 in the same figure except that the primary antibodies were replaced with normal rabbit serum.

**Figure 4:**
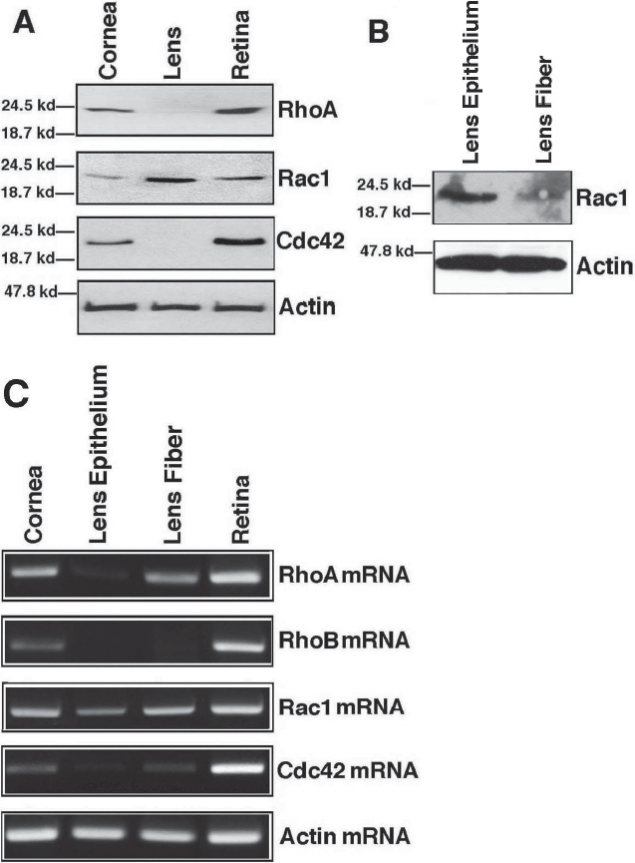
Western blot and reverse transcription polymerase chain reaction analysis of the expression patterns of RhoA, Rac1, and Cdc42 in different compartments of the adult mouse eye. **A**: Western blot analysis of RhoA, Rac1, and Cdc42 in cornea, lens, and retina. Note the differential expression patterns of three small GTPases in cornea, lens, and retina. While only Rac1 was expressed in the lens, all three small GTPases were expressed in both cornea and retina. **B**: Western blot analysis of Rac1 in lens epithelium and fiber cells. Expression of Rac1 is predominant in epithelium but much less so in lens fiber cells. **C**: RT-PCR analysis of RhoA, RhoB, Rac1 and Cdc42 in cornea, lens epithelium, lens fibers and retina. Note that the mRNA for the four small GTPases were all expressed in relatively high levels in retina and slightly lower levels in the cornea. In the lens, while Rac1 was expressed in both epithelial cells and fiber cells, only traces of RhoA and Cdc42 mRNA were detected in the epithelium. A relatively high level of RhoA mRNA was detected in the lens fiber cells. However, RhoA protein is not detectable, indicating its translational control. Similarly, some Cdc42 mRNA was also detectable in the fiber cells although Cdc42 protein is not detected. RhoB mRNA is absent in both epithelium and fiber cells of the adult lens.

**Figure 5:**
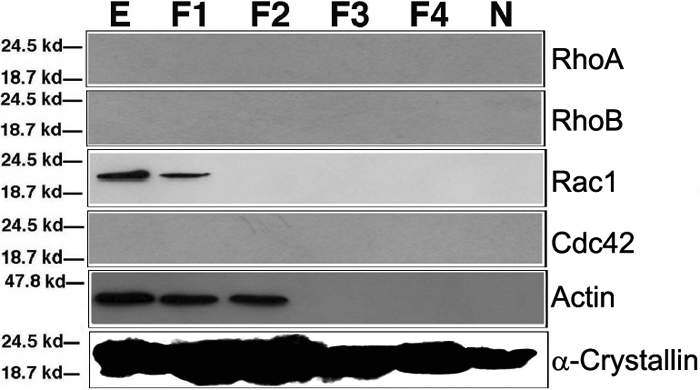
Western blot analysis of the expression patterns of RhoA, RhoB, Rac1, and Cdc42. from four-week-old rat lens as determined by western blot analysis lens epithelium (E), cortical fiber cells (F1), subcortical fiber cells (F2), middle layer of fiber cells (F3), inner layers of fiber cells (F4), and nuclear fiber cells (N) from four-week-old rat lens as determined by western blot analysis. As comparison, the expression patterns of β-actin and α-crystallin were also included. Note that as observed in the mouse lens, Rac1 was expressed mainly in the lens epithelium and to a lesser degree in F1 lens fiber cells. β-Actin was detectable in the lens epithelium, cortical (F1), and sub-cortical (F2) fiber cells. In contrast, α-crystallin is present in both lens epithelium and all layers of lens fiber cells.

Compared with the control, the background staining of RhoA and Cdc42 in the nuclei of the epithelial and fiber cells were stronger (Figure 3A, the leftmost panel, and the rightmost two panels), however, western blot analysis confirmed their absence in the adult mouse lens (Figure 4A). Rac1 expression, however, was found exhibiting a strong level of expression throughout the lens, more so in the epithelial cells than in the fiber cells (Figure 3; the second panel from the left). The strong expression of Rac1 in the ocular lens of the adult mouse eye was further confirmed by Western blot analysis (Figure 4A). At the mRNA level, RT-PCR analysis demonstrated that Rac1 mRNA was strongly expressed in both epithelial and fiber cells (Figure 4C). However, for RhoA and Cdc42 mRNAs, they were easily detected in the fiber cells but barely detected in the epithelial cells (Figure 4C). The exclusive expression of Rac1 in the eye lens was also confirmed in rat lens by western blot analysis (Figure 5). Analysis of the three small GTPases in the epithelial cells (E), cortical fiber cells (F1), subcortical fiber cells (F2), middle layer of fiber cells (F3), inner layer of fiber cells (F4), and primary fiber cells (N) of four-week old rat lens revealed that only Rac1 was present in the adult lens (Figure 5). A further analysis of Rac1 expression in the mouse lens revealed that much stronger expression of Rac1 was found in the epithelial cells than in the fiber cells (Figure 4B), which was a contrast to the mRNA distribution for Rac1 in the two types of cells of the mouse lens (Figure 4C). In addition, Rac1 expression was only detected in the cortical fiber cells among the different layers of fibers (Figure 5).

### Expression of RhoA, Rac1, and Cdc42 in the cornea of the adult mouse eye

Immunohistochemistry analysis demonstrated that the highest level of RhoA expression in the cornea of the adult mouse eye was detected in the endothelial cells and the surface layer of epithelial cells (Figure 3B; leftmost panel) with little detectable staining in the columnar cells of the corneal epithelium. In the corneal stroma, a strong staining was detected in the bodies of some fibroblasts (Figure 3B; leftmost panel). The fluorescence signal outside of the fibroblasts reflects non-specific binding (Figure 3B). In contrast, Rac1 was predominantly expressed in the entire layers of corneal epithelium (both columnar and squamous cell layers) but absent in both corneal stroma and the endothelial cells layer (Figure 3B; the second panel from the left). For cdc42, immunocytochemistry demonstrated that it was expressed in both the endothelial cell layer and the entire layers of corneal epithelium (both columnar and squamous cell layers) but not in the corneal stroma (Figure 3B, the third panel from the left). To further confirm the expression of RhoA, Rac1, and Cdc42 in the cornea of the adult mouse eye, western blot analysis was conducted. As shown in Figure 4A, all three small molecule GTPases were detected in the cornea of the adult mouse eye. RT-PCR analysis revealed that the mRNAs coding for RhoA, Rac1, and Cdc42 were also present in the cornea of adult mouse eye (Figure 4C).

### Expression of RhoA, Rac1 and Cdc42 in the retina of the adult mouse eye

In the retina of the adult mouse eye, immunohistochemistry showed that both RhoA and Cdc42 were highly expressed in the photoreceptors, the horizontal/amacrine/Muller cell layers, and in some of the ganglion cells (Figure 3C). In most ganglion cells (the inner and outer plexiform layers), expression of the small GTPases was detected, but on a much reduced level (Figure 3C). Compared with RhoA and Cdc42, expression of Rac1 was lower. This expression pattern of RhoA, Rac1, and Cdc42 in the retina of the adult mouse eye was further confirmed by western blot analysis (Figure 4A) and RT-PCR (Figure 4C).

## Discussion

### Differential expressions of RhoA, Rac1, and Cdc42 in the cornea of mouse eye suggest their roles in corneal differentiation and maturation

The Rho GTPase family contains about 18 members and among them, RhoA, Rac1, and Cdc42 have been characterized most extensively [7,8,17,18] These small GTPases are best known for their effects on the actin cytoskeleton. Their effects include reorganization of the actin cytoskeleton into distinct structures such as stress fibers and focal adhesions, veil-like lamellipodia, and filopodial microspikes [7,8,17,18]. In addition, these small GTPases have been shown to play a role in transcriptional activation, membrane trafficking, and microtubule dynamics, all of which contribute to regulation of cell growth, cytokinesis, cell motility, cell-cell interactions, cell-extracellular matrix adhesion, cell transformation, and invasion [7,8,17,18].

During mouse eye development, a two-cell-layer corneal peridermal epithelium forms when the lens vesicle becomes detached from the surface ectoderm at E11.5 pc. This peridermal epithelium differentiates into three parts of the cornea: the endothelium containing a single layer of cells facing the aqueous humor, the epithelium consisting of numerous layers of epithelial cells, and the stroma containing majority of collagen fibers and some fibroblasts [19]. During the morphogenetic process of corneal formation, cell proliferation, growth, migration, and differentiation are frequently occurring, thus it is conceivable that the functions of small GTPases are necessary. Our demonstration that low levels of RhoA, Rac1, and Cdc42 are all present in the embryonic cornea supports our speculation that these small GTPases play a role in corneal differentiation. On the other hand, RhoB has been reported negative in the cornea during embryonic development of the mouse eye [20]. This is not surprising considering that RhoB is in many cases growth-inhibiting [21,22] and has been shown to be down-regulated in cancer cells [23].

It has been shown that mouse corneal differentiation begins at E15.5 when keratin 12 is expressed in suprabasal cells of the peridermal cornea [24]. This differentiation process continues after birth and corneal maturation does not occur until about three to six months after birth [24]. Consistent with corneal growth and differentiation, we found that expression of the three small GTPases are gradually enhanced in the cornea of the postnatal mouse eye and seems to peak in the cornea of adult mouse eye (Figure 2 and Figure 3B). Additionally, in the postnatal mouse cornea, Rac1 displayed the highest level of expression, RhoA the intermediate level, and Cdc42 the lowest level in both epithelial and endothelial cells of the cornea. Such differential expression patterns suggest that the three small GTPases may play different roles during corneal differentiation.

The adult cornea contains a stratified epithelium in which the superficial corneal epithelial cells slough off as a result of terminal differentiation. The corneal epithelial cells are then replenished by the division of basal cells [25,26]. We have demonstrated that in the adult mouse, all three small GTPases are expressed in the corneal epithelium. However, while RhoA is expressed in the superficial epithelial cells, the other small GTPases, Rac1 and Cdc 42, are expressed in the entire epithelial layers of cells. These results suggest that RhoA, Rac1, and Cdc42 may all be involved in terminal differentiation but only Rac1 and Cdc 42 seems to be necessary for the process of corneal epithelial self-renewal. In contrast, the exclusive expression of RhoA in the endothelial cells indicates its unique role in this part of the cornea. The presence of RhoA but not Rac1 or Cdc42 in the fibroblasts of the stroma of the adult cornea suggests that only RhoA is necessary for the active functions of the fibroblasts. In summary, our results reveal the first defined patterns of differential expression of the three small molecule GTPases in mouse cornea, which provides the basis for understanding their roles in regulating corneal differentiation and formation.

### While RhoA, Rac1, and Cdc42 all seem to be important for embryonic lens development, Rac1 alone is involved in adult lens growth and differentiation

The early immunohistochemistry analysis of RhoB expression pattern revealed its possible important role during lens morphogenesis. RhoB was strongly expressed in the developing lens, specifically paralleling the morphogenesis of the lens fiber cells [20]. In the present study, we detected a similar expression pattern of RhoA in mouse embryonic lens from 11.5 pc to 17.5 pc (Figure 1), which suggests involvement of the RhoA in lens vesicle formation and differentiation. Different from both RhoA and RhoB, expression of Cdc42 was lower at the lens vesicle stage (11.5 pc) and definitive expression of Rac1 was only detected at 14.5 pc. This finding implies that the function of both Rac1 and Cdc42 comes into play at a later stage. This is not surprising considering that both RhoA and RhoB are involved in the regulation of actin stress fibers and of cell contacts thus the functions of the above two small GTPases are necessary during lens vesicle formation when cell proliferation plays a dominant role. Alternatively, during the differentiation of the primary lens fiber cells, these fiber cells require massive morphogenetic movements where filopodia formation and membrane ruffling are frequently observed. The expression of Cdc42 and Rac1 at the beginning of lens fiber differentiation illustrates their active participation in this differentiation process. At the stage of 17.5 pc, RhoA, RhoB, Rac1, and Cdc42 are all strongly expressed. This is consistent with the fact that both cell division and lens cell differentiation occur at the same time. In the postnatal lens, cell division in the germinal zone continues and the differentiating lens cells migrate into the equatorial region and differentiate into secondary fiber cells. At this stage, the lens is still in the very active growth phase so expression of all four GTPases are still strong (Figure 2 and [20]). However, in the adult lens, the growth is substantially slowing down and the lens differentiation takes a much longer time. Consistent with this slow growth, expression of RhoA and Cdc42 are downregulated to an undetectable level in both the mouse lens (Figure 4) and the rat lens (Figure 5). Rac1 is likely the major small GTPase responsible for adult lens growth.

In the present study, we observed in the adult lens that the proteins for RhoA and Cdc42 are undetectable, however, the mRNAs for these small GTP proteins are easy detectable in the fiber cells, although their expressions were just barely detectable in the epithelial cells.

Considering the fiber cells are in different stages of differentiation and the differentiating fiber cells are focusing their energy and metabolism to disassemble all the cellular organelles, mRNAs in the differentiation fiber cells are likely either untranslatable or translatable in low efficiency. Consistent with this hypothesis is the demonstration of the amount Rac1 mRNA in fiber cells of mouse lens being higher than in epithelial cells. However, the Rac1 protein in the fiber cells of mouse lens is lower than that in epithelial cells (Figure 4B,C). Moreover, our previous studies have revealed that the mRNA for the catalytic subunit of PP-2A is present in cortical layers of fiber cells of rat lens. However, the protein for the catalytic subunit of PP-2A is undetectable [12]. As a result, while the mRNAs for RhoA and Cdc42 are unsurprisingly detectable in mouse lens but their proteins are absent. Our results of the absence of RhoB mRNA in the adult mouse is consistent with the early work by Maddala et al. [20].

### RhoA, Rac1, and Cdc42 all seem to be involved in control of retina differentiation during mouse eye development

In the present study, we have demonstrated that the three small GTPases, RhoA, Rac1, and Cdc42 are all expressed during embryonic retina. While, at the 11.5 pc and 14.5 pc, RhoA and Cdc42 are relatively stronger than Rac1, the opposite is true for their expressions at 17.5 pc. The expression levels of all three small GTPases were increased to maximum in the postnatal mouse eye retina. This is consistent with the fact that during and after the birth period, active mouse retinal differentiation occurs while retina maturation does not occur until several weeks after birth. Once the retina becomes mature, the expression levels of the three small GTPases become substantially decreased, further proving their role in differentiation (Figure 3). From embryonic stage to adult mouse eye, it was found that the strongest retina expression of all three small GTPases were found in photoreceptors then in the nuclear layer and some of the ganglion cells while the weakest expression was observed in both outer and inner plexiforms.

During retinal development, Rho-GTPases seem to play an essential role in regulating neuritogenesis and controlling neural cell growth, differentiation, guidance, and branching. They have been found to regulate similar functions in Xenopus, Drosophila, and mammals [27-29].

In comparison to the expression patterns of these three GTPases in chicken retina [6], some differences of the expression patterns between chicken and mouse retina are revealed. First, in the chicken embryonic retina, the three small GTPases, RhoA, Rac1, and Cdc42, were initially expressed at very high levels in the ganglion cells while in the mouse retina, they were strongly expressed in the photoreceptors of the retina. Second, during the embryonic stage, strong levels of RhoA and Cdc42 were detected in the inner plexiform of chicken retina [6] but they were not detected in the mouse retina. Additionally, Cdc42 appears to display a stronger expression level than RhoA in the chicken retina. On the contrary, RhoA appears to display a stronger expression signal than Cdc42 in the mouse retina. The expression patterns of the small GTPases in the two types of vertebrates also show substantial similarities. Like the postnatal retina of chicken, the immunoreactive signal for Rac1 in the adult mouse retina was the lowest (Figure 3 and [6]). It is also generally true that the three small GTPases are strongly expressed in photoreceptors, the cell bodies of horizontal/amacrine/Muller cells in the nuclear layer, and some ganglion cells, but are weakly expressed in the outer plexiform layer.

In conclusion, previous studies have shown that the Rho-family of GTPases is essential in modulating cytoskeletal rearrangements as well as mediating adhesive and morphological properties of the cell, transcriptional regulation, and differentiation [30,31]. For this reason, it is not surprising that RhoA, Rac1, and Cdc42 are all highly expressed during mouse ocular tissue development and continue to be specifically expressed in various regions of the adult eye. Although RhoA is known to function in opposition to Rac1 and Cdc42-mediated activity in many cell systems, it is likely that the presence of each is essential during eye development. Our expression analysis of the adult mouse showed that each of these Rho-GTPases is isolated to discrete regions of the adult eye suggesting that their presence is necessary for the maintenance and normal function of these areas.

## References

[1] Ridley AJ Hall A The small GTP-binding protein rho regulates the assembly of focal adhesions and actin stress fibers in response to growth factors. Cell 1992 70 389 99 1643657 10.1016/0092-8674(92)90163-7

[2] Zipkin ID Kindt RM Kenyon CJ Role of a new Rho family member in cell migration and axon guidance in C. elegans. Cell 1997 90 883 94 9298900 10.1016/s0092-8674(00)80353-0

[3] Mackay DJ Hall A Rho GTPases. J Biol Chem 1998 273 20685 8 9694808 10.1074/jbc.273.33.20685

[4] Hall A Rho GTPases and the actin cytoskeleton. Science 1998 279 509 14 9438836 10.1126/science.279.5350.509

[5] O'Kane EM Stone TW Morris BJ Activation of Rho GTPases by synaptic transmission in the hippocampus. J Neurochem 2003 87 1309 12 14622110 10.1046/j.1471-4159.2003.02102.x

[6] Santos-Bredariol AS Santos MF Hamassaki-Britto DE Distribution of the small molecular weight GTP-binding proteins Rac1, Cdc42, RhoA and RhoB in the developing chick retina. J Neurocytol 2002 31 149 59 12815236 10.1023/a:1023997506760

[7] Luo L Rho GTPases in neuronal morphogenesis. Nat Rev Neurosci 2000 1 173 80 11257905 10.1038/35044547

[8] Govek EE Newey SE Van Aelst L The role of the Rho GTPases in neuronal development. Genes Dev 2005 19 1 49 15630019 10.1101/gad.1256405

[9] Rao PV Zigler JS Jr Garland D Analysis of small GTP-binding proteins of the lens by GTP overlay assay reveals the presence of unique GTP-binding proteins associated with fiber cells. Exp Eye Res 1997 64 219 27 9176056 10.1006/exer.1996.0197

[10] Brennan WA Jr, Lin SH. Solubilization and purification of the rat liver insulin receptor. In: Marshak DR, Kadonaga JT, Burgess RR, Knuth MW, Brennan WA, Lin SH. Strategies for Protein Purification and Characterization: A Laboratory Course Manual. New York: Cold Spring Harbor Press; 1996. p. 295-346.

[11] Spector DL, Goldman RD, Leinwand L, eds. Cells: A Laboratory Manual. New York: Cold Spring Harbor Laboratory Press; 1997. p. 34.1-35.14.

[12] Li DW Xiang H Fass U Zhang XY Analysis of expression patterns of protein phosphatase-1 and phosphatase-2A in rat and bovine lenses. Invest Ophthalmol Vis Sci 2001 42 2603 9 11581206

[13] Li DW Liu JP Wang J Mao YW Hou LH Expression and activity of the signaling molecules for mitogen-activated protein kinase pathways in human, bovine, and rat lenses. Invest Ophthalmol Vis Sci 2003 44 5277 86 14638727 10.1167/iovs.03-0348

[14] Peterson GL A simplification of the protein assay method of Lowry et al. which is more generally applicable. Anal Biochem 1977 83 346 56 603028 10.1016/0003-2697(77)90043-4

[15] Wang J Feng H Huang XQ Xiang H Mao YW Liu JP Yan Q Liu WB Liu Y Deng M Gong L Sun S Luo C Liu SJ Zhang XJ Liu Y Li DW Human telomerase reverse transcriptase immortalizes bovine lens epithelial cells and suppresses differentiation through regulation of the ERK signaling pathway. J Biol Chem 2005 280 22776 87 15849192 10.1074/jbc.M500032200

[16] Li DW Liu JP Schmid PC Schlosser R Feng H Liu WB Yan Q Gong L Sun SM Deng M Liu Y Protein serine/threonine phosphatase-1 dephosphorylates p53 at Ser-15 and Ser-37 to modulate its transcriptional and apoptotic activities. Oncogene 2006 25 3006 22 16501611 10.1038/sj.onc.1209334

[17] Van Aelst L D'Souza-Schorey C Rho GTPases and signaling networks. Genes Dev 1997 11 2295 322 9308960 10.1101/gad.11.18.2295

[18] Burridge K Wennerberg K Rho and Rac take center stage. Cell 2004 116 167 79 14744429 10.1016/s0092-8674(04)00003-0

[19] Zieske JD Corneal development associated with eyelid opening. Int J Dev Biol 2004 48 903 11 15558481 10.1387/ijdb.041860jz

[20] Maddala R Peng YW Rao PV Selective expression of the small GTPase RhoB in the early developing mouse lens. Dev Dyn 2001 222 534 7 11747086 10.1002/dvdy.1202

[21] Du W Lebowitz PF Prendergast GC Cell growth inhibition by farnesyltransferase inhibitors is mediated by gain of geranylgeranylated RhoB. Mol Cell Biol 1999 19 1831 40 10022870 10.1128/mcb.19.3.1831 PMC83976

[22] Chen Z Sun J Pradines A Favre G Adnane J Sebti SM Both farnesylated and geranylgeranylated RhoB inhibit malignant transformation and suppress human tumor growth in nude mice. J Biol Chem 2000 275 17974 8 10770919 10.1074/jbc.C000145200

[23] Adnane J Seijo E Chen Z Bizouarn F Leal M Sebti SM Munoz-Antonia T RhoB, not RhoA, represses the transcription of the transforming growth factor beta type II receptor by a mechanism involving activator protein 1. J Biol Chem 2002 277 8500 7 11741970 10.1074/jbc.M104367200

[24] Tanifuji-Terai N Terai K Hayashi Y Chikama T Kao WW Expression of keratin 12 and maturation of corneal epithelium during development and postnatal growth. Invest Ophthalmol Vis Sci 2006 47 545 51 16431949 10.1167/iovs.05-1182

[25] Kao WW Liu CY The Use of Transgenic and Knock-out Mice in the Investigation of Ocular Surface Cell Biology. Ocul Surf 2003 1 5 19 17075625 10.1016/s1542-0124(12)70003-4

[26] Sun TT Lavker RM Corneal epithelial stem cells: past, present, and future. J Investig Dermatol Symp Proc 2004 9 202 7 15369214 10.1111/j.1087-0024.2004.09311.x

[27] Yuan XB Jin M Xu X Song YQ Wu CP Poo MM Duan S Signalling and crosstalk of Rho GTPases in mediating axon guidance. Nat Cell Biol 2003 5 38 45 12510192 10.1038/ncb895

[28] Ruchhoeft ML Ohnuma S McNeill L Holt CE Harris WA The neuronal architecture of Xenopus retinal ganglion cells is sculpted by rho-family GTPases in vivo. J Neurosci 1999 19 8454 63 10493746 10.1523/JNEUROSCI.19-19-08454.1999 PMC6783015

[29] Settleman J Rac 'n Rho: the music that shapes a developing embryo. Dev Cell 2001 1 321 31 11702944 10.1016/s1534-5807(01)00053-3

[30] Hill CS Wynne J Treisman R The Rho family GTPases RhoA, Rac1, and CDC42Hs regulate transcriptional activation by SRF. Cell 1995 81 1159 70 7600583 10.1016/s0092-8674(05)80020-0

[31] Van Aelst L Symons M Role of Rho family GTPases in epithelial morphogenesis. Genes Dev 2002 16 1032 54 12000787 10.1101/gad.978802

